# Review of *Dibrachys* Förster from China (Hymenoptera, Chalcidoidea, Pteromalidae)

**DOI:** 10.3897/zookeys.656.11373

**Published:** 2017-02-14

**Authors:** Tian-yang Jiao, Qin-ying Yao, Hui Xiao

**Affiliations:** 1 Key Laboratory of Zoological Systematics and Evolution, Institute of Zoology, Chinese Academy of Sciences, Beijing, 100101, P. R. China; 2 College of Life Sciences, University of Chinese Academy of Sciences, Beijing, 100101, P. R. China; 3 College of Life Science and Technology, Hebei University, Baoding, 071002, P. R. China

**Keywords:** China Mainland, *Dibrachys*, key, new species, new record, Pteromalidae, taxonomy

## Abstract

Twelve species of *Dibrachys* Förster are studied from China, of which four new species, *Dibrachys golmudica* Jiao & Xiao, **sp. n.**, *Dibrachys kunmingica* Jiao & Xiao, **sp. n.**, *Dibrachys liaoi* Jiao & Xiao, **sp. n.** and *Dibrachys qinghaiensis* Jiao & Xiao, **sp. n.**, and four newly recorded species, *Dibrachys braconidis* (Ferrière & Faure), *Dibrachys confusus* (Girault), *Dibrachys hians* Bouček and *Dibrachys maculipennis* Szelényi, are reported. A key to Chinese *Dibrachys* and illustrations of external features of the species are provided.

## Introduction

*Dibrachys* was erected by Förster in 1856, but without any species included in the genus. Thomson (1878) subsequently designated *Pteromalus boucheanus* Ratzeburg, 1844 as the type species and listed *Pteromalus cavus* Walker, 1835 as a synonym. Although the type material of *Pteromalus boucheanus* is lost (Graham 1969), Thomson’s work was accepted by the majority of later researchers. Graham (1969) designated a lectotype for *Pteromalus cavus* and suggested that, failing the discovery of the type material of *Pteromalus boucheanus*, the lectotype of *Pteromalus cavus* might conveniently be made also the neotype of *Pteromalus boucheanus* because the two were supposed to be identical. However, Peters and Baur (2011) designated a different specimen as lectotype as part of their review of the *Dibrachys cavus* species complex, in which they treated *Dibrachys cavus* as a junior synonym of *Dibrachys microgastri* (Bouché, 1834). Consequently, *Dibrachys microgastri* (Bouché) is the senior synonym of both *Dibrachys boucheanus* (Ratzeburg) and *Dibrachys cavus* (Walker).

Based on differences in mandibular formula and fore wing marginal fringe, Bouček (1965) divided the genus into two subgenera *Dibrachys* Förster s. str. and *Dibrachys* (*Allodibrachys* Bouček). Nineteen valid species of *Dibrachys* are recognized, of which 13 are known from the Palearctic region, 8 from the Nearctic region, 4 from the Oriental region, 1 from the Australasian region, 1 from the Afrotropical region and 2 from the Neotropical region (Noyes 2016). Most species are parasitoids of insect pests, and play an important role in biological control, with 372 different host species being reported for *Dibrachys* (Grissell 1974; Noyes 2016), including species of Lepidoptera, Hymenoptera, Diptera, Coleoptera, Dermaptera, Hemiptera, Neuroptera, Strepsiptera, and several species of Arachnida (Araneidae and Philodromidae). However, as Graham (1969) noted, almost all the host records are associated with *Dibrachys cavus*.

Only four species of *Dibrachys* have previously been recorded in China (Liao 1987; Yang 1996). In this study 12 species are identified from China, including four new species (*Dibrachys golmudica* Jiao & Xiao, sp. n., *Dibrachys kunmingica* Jiao & Xiao, sp. n., *Dibrachys liaoi* Jiao & Xiao, sp. n., *Dibrachys qinghaiensis* Jiao & Xiao, sp. n.) and four newly recorded species (*Dibrachys braconidis* (Ferrière & Faure), *Dibrachys confusus* (Girault), *Dibrachys hians* Bouček, and *Dibrachys maculipennis* Szelényi).

## Material and methods

A total of 943 specimens was examined from the museum of Institute of Zoology, the Chinese Academy of Sciences (IZCAS). All material from our own collection was swept and preserved in 75% ethanol. Specimens were subsequently air dried, point-mounted and examined with a Nikon SMZ1500 stereomicroscope. Photographs were taken using a Nikon Multizoom AZ100 system, and the plates were compiled using Adobe Photoshop CS3 software. In addition, the author had examined specimens of *Dibrachys* deposited in the National History Museum, London, the Naturalis, Leiden and the Bavarian State Collections of Zoology in April, 2002.

Morphological terminology mostly follows that of Graham (1969), Bouček (1988) and Gibson (1997). All specimens were examined and identified using the keys of Graham (1969), Grissell (1974), Doganlar (1987), Yang (1996), Zerova et al (1992) and Peters and Baur (2011). Every new species is described based on the holotype specimen, other species are described basing on the examined material available to us. Body length excludes the ovipositor sheaths and is measured in millimeters (mm); other measurements are given as ratios.

Abbreviations of morphological terms used are:

Fun
 funicular segment number 


POL
 posterior ocellar distance 


OOL
 ocellocular distance 


Gtn
 gastral tergum number 


## Taxonomy

### Key to species

**Table:** 

1	Left mandible with three teeth and right mandible with four teeth (Fig. 6); fore wing with marginal fringe except between marginal vein and wing apex; occipital carina transverse, closer to foramen than vertex; gaster mostly ovate (Figs 1, 5), slightly longer than broad	**Dibrachys (Allodibrachys)**...**2**
–	Both mandibles with four teeth (Fig. 21); fore wing without marginal fringe; occipital carina curving, closer to vertex than foramen; gaster spindle-shaped (Figs 15, 23, 33, 38), distinctly longer than broad	**Dibrachys (Dibrachys)**...**6**
2	Head in frontal view with gena almost straight and with lower angle of gena protruding beyond clypeal margin (Fig. 3)	***Dibrachys hians***
–	Head in frontal view with gena evenly curved and with lower angle of gena not exceeding clypeal margin (Figs 6, 12)	**3**
3	Stigmal vein longer than postmarginal vein; gaster 1.5 times as long as broad	***Dibrachys koraiensis***
–	Stigmal vein shorter than or at most as long as postmarginal vein; gaster at most 1.3 times as long as broad	**4**
4	Lower margin of clypeus not protruded, slightly emarginate in middle and without tooth (Fig. 6); head in dorsal view with POL 1.33 times as long as OOL	***Dibrachys kojimae***
–	Lower margin slightly protruded, emarginate in middle and with two obtuse teeth; head in dorsal view with POL more than 1.5 times as long as OOL	**5**
5	Marginal vein 1.5 times as long as stigmal vein; propodeum with plica complete and median carina only distinct basally	***Dibrachys yunnanensis***
–	Marginal vein 1.91 times as long as stigmal vein; propodeum with plica only conspicuous basally, median carina absent	***Dibrachys kunmingica* sp. n.**
6	Antennae with three anelli; postmarginal vein very shorter than stigmal vein, at most half length of stigmal vein	***Dibrachys golmudica* sp. n.**
–	Antennae with two anelli; postmarginal vein longer or slightly shorter than stigmal vein	**7**
7	Lower margin of clypeus broadly emarginate, without tooth; gaster 1.37 times as long as broad	***Dibrachys braconidis***
–	Lower margin of clypeus slightly protruding, emarginate in middle and with two blunt or sharp teeth; gaster at least 1.8 times as long as broad	**8**
8	Antennal insertion slightly above lower ocular line, antennal scape reaching lower margin of anterior ocellus	**9**
–	Antennal insertion place on lower ocular line, antennal scape not reaching lower margin of anterior ocellus	**11**
9	Fore wing with a yellowish-brown infumation behind marginal vein; propodeum with incomplete median carina; stigmal vein slightly longer than postmarginal vein; gaster slightly broader than thorax width	***Dibrachys maculipennis***
–	Fore wing immaculate, without any infumation; propodeum with complete median carina; stigmal vein as long as postmarginal vein; gaster narrower than thorax width	**10**
10	Antennal scape as long as eye height; head in frontal view 1.27 times as wide as high	***Dibrachys qinghaiensis* sp. n.**
–	Antennal scape distinctly shorter than eye height; head in frontal view 1.15 times as wide as high	***Dibrachys confusus***
11	Lower margin of clypeus emarginate in middle and with two sharp teeth (Fig. 31); antennae with Fu_1_ to Fu_4_ slightly longer than its broad; head in dorsal view 1.8 times as wide as long; gaster 1.8 times as long as broad	***Dibrachys liaoi* sp. n.**
–	Lower margin of clypeus emarginate in middle and with two blunt teeth (Figs 40, 45) ; antennae with Fu_1_ to Fu_4_ quadrate; head in dorsal view 2 times as wide as long; gaster 2 times as long as broad	***Dibrachys microgastri***

#### Dibrachys

Taxon classificationAnimaliaHymenopteraPteromalidae

Förster, 1856

Dibrachys Förster, 1856: 65. Type-species: Pteromalus boucheanus Ratzeburg, designated by Thomson 1878: 47 (= Diplolepis microgastri Bouché, 1834: 168).
Dibrachys Förster: Dalla Torre 1898: 155; Graham 1969: 804–814; Wallace 1973: 175–176; Bouček 1993: 1259; Yang 1996: 196–201, 323–324.
Coelopisthoidea Gahan, 1913: 178–183. Type-species: Coelopisthoidea cladiae Gahan, 1913: 178–183. Synonymized by Girault 1916b: 408; Bouček 1988: 434.

##### Diagnosis.

Body dark green. Head in frontal view round; antennal insertion placed on lower ocular line and face not protuberant at antennal insertion; antennal formula 11263 (rarely 11353); lower margin of clypeus with two sinuate teeth; both mandibles with four teeth or right mandible with four teeth and left mandible with three teeth; head in dorsal view with occiput margined by blunt or sharp, transverse ridge. Mesosoma slightly convex; pronotal collar not margined or slightly carinate medially; notauli incomplete and inconspicuous; scutellum without frenal groove; propodeum with plica complete, median carina developed or not. Fore wing without marginal fringe or at least bare between postmarginal vein and wing apex; postmarginal vein short, only inconspicuously longer than stigmal vein. Hind tibia with one spur. Gaster ovate.

##### Distribution.

Widespread world-wide distribution, see Noyes (2016). China: Heilongjiang, Jilin, Liaoning, Inner Mongolia, Beijing, Hebei, Shanxi, Shandong, Henan, Shaanxi, Ningxia, Gansu, Qinghai, Xinjiang, Jiangsu, Shanghai, Anhui, Zhejiang, Hubei, Jiangxi, Hunan, Guangxi, Sichuan, Guizhou, Yunnan and Tibet.

### Descriptions of species

#### Dibrachys (Allodibrachys)

Taxon classificationAnimaliaHymenopteraPteromalidae

Bouček

Dibrachys sgen. Allodibrachys Bouček, 1965: 30. Type-species : Dibrachys hians Bouček, by original designation.

##### Diagnosis.

The subgenus have the left mandible with three teeth and right mandible with four teeth; occipital carina transverse, closer to foramen than vertex; fore wing with marginal fringe except between marginal vein and wing apex; gaster mostly ovate, slightly longer than broad.

#### Dibrachys hians

Taxon classificationAnimaliaHymenopteraPteromalidae

Bouček, 1965, new record to China

Figs 1–4

Dibrachys (Allodibrachys) hians Bouček, 1965: 28.

##### Diagnosis.

**Figures 1–9.**
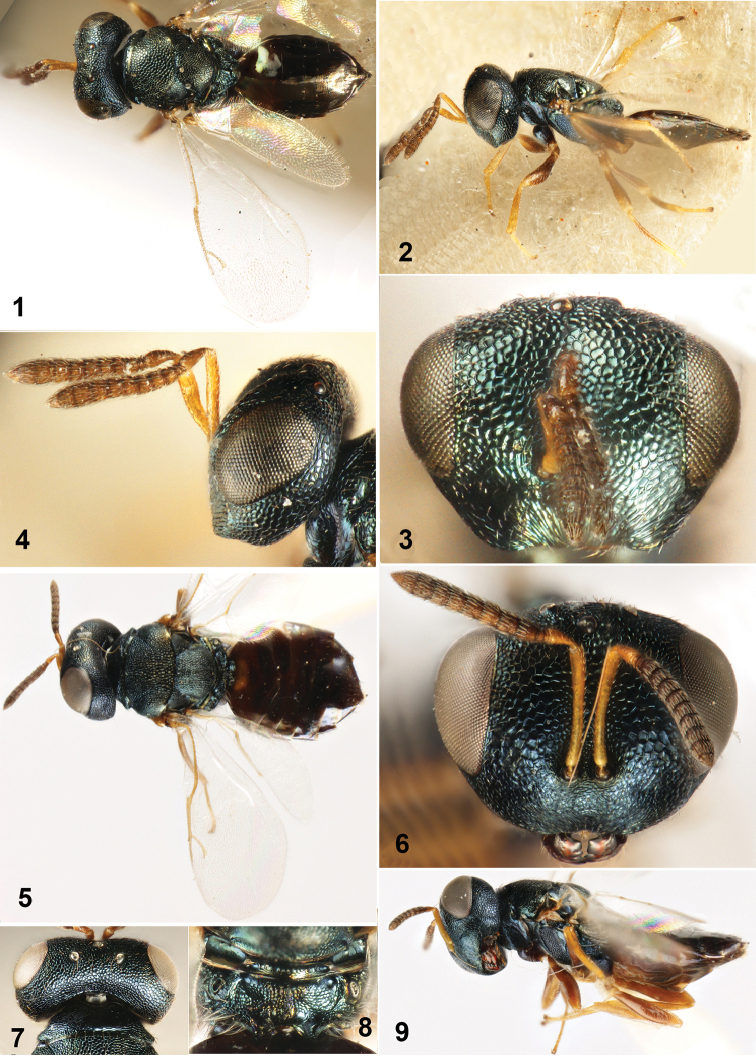
**1–4** *Dibrachys hians* Bouček, 1965. **1** Body in dorsal view **2** Body in lateral view **3** Head in frontal view **4** Head in lateral view **5–9** *Dibrachys kojimae* (Ishii, 1916) **5** Body in dorsal view **6** Head in frontal view **7** Head in dorsal view **8** Propodeum **9** Body in lateral view.

Body length 1.6-2.0 mm (Figs 1, 2). Head in frontal view (Fig. 3) 1.32 × as wide as high; antennal scrobe shallow, extending upwards and not reaching anterior ocellus; antennal insertion placed on lower ocular line; clypeus with longitudinal striation; lower margin of clypeus not protruded, emarginate in middle, and without tooth; gena almost straight, lower angle of gena protruding beyond clypeal margin. Antenna (Fig. 4) with scape shorter than eye height (0.79×), not reaching anterior ocellus; length of pedicel and flagellum combined less than head width (0.67×); anelli transverse; each funicular segment subquarate; clava slightly clavate. Head in dorsal view with width 2× length; eye length 2× temple length; POL 2× OOL. Mesosoma 1.37× as long as broad, mid lobe of mesoscutum with regular sculpture. Propodeum with complete median carina and incomplete plica. Fore wing with marginal vein 1.8× as long as postmarginal vein; postmarginal vein as long as stigmal vein. Gaster 1.25× as long as broad, slightly broader than thorax width.

##### Material examined.

China: 3♀, Heilongjiang: Yichun, 13.VII.1962, ex. Tachinidae sp. on *Ptycholomoides aeriferanus* Herrich-Schaffer, leg. Ding-Xi Liao; 1♂, 1♀, Heilongjiang: Dailing, 29.VI.1962, ex. Tachinidae sp. on *Ptycholomoides aeriferanus* Herrich-Schaffer; 1♀, Jilin: Dunhua, 25.VI.1990; 1♂, 2♀, Beijing: Yanqing, 19.VII.1982, ex. Curculionidae sp. on elm, leg. Ding-Xi Liao.

##### Hosts.

Trematerra (1988) reported *Pyralis farinalis* L (Lepidoptera: Pyralidae). as a host and here we record Curculionidae sp. on elm, Tachinidae sp. on *Ptycholomoides aeriferanus* Herrich-Schaffer (Lepidoptera: Tortricidae), and Tachinidae sp. on *Laspeyresia grunertiana* (Ratzeburg) (Lepidoptera: Noctuoidea).

##### Distribution.

China (Heilongjiang, Jilin, Beijing); Palearctic and Nearctic regions.

#### Dibrachys kojimae

Taxon classificationAnimaliaHymenopteraPteromalidae

(Ishii, 1938)

Figs 5–9

Euterus kojimae Ishii, 1938: 100.
Dibrachys kojimae (Ishii): Kamijo 1982: 74.

##### Diagnosis.

Body length 2.5–3.2 mm (Figs 5, 9). Head in frontal view (Fig. 6) 1.31× as wide as high; sculpture on lateral area of antennal scrobe distinctly larger than on vertex and face; lower margin of clypeus not protruded, slightly emarginate in median; gena evenly curved, lower angle of gena not exceeding clypeal margin. Antenna with scape shorter than eye height (0.81×); length of pedicel and flagellum combined less than head width (0.94×); anelli transverse and second anellus longer than first anellus; Fu_1_-Fu_3_ slightly long than wide, Fu_4_-Fu_6_ subquadrate. Head in dorsal view (Fig. 7) 2× as wide as long; eye length 1.67× temple length, POL 1.33× OOL. Mesosoma 1.32× as long as broad; with regular sculpture. Propodeum (Fig. 8) with incomplete median carina and complete plica. Fore wing with submarginal vein 2.3× as long as marginal vein; marginal vein 1.6× as long as postmarginal vein, 1.8× as long as stigma vein; stigmal vein slightly shorter than postmarginal vein (0.9×). Gaster 1.2× as long as broad, slightly broader than thorax width.

##### Material examined.

China: 2♂, 16♀, Beijing: Miyun Reservoir, 10-20.VII.1983, ex. pupae of *Dendrolimus tabulaeformis* Tsai *et* Liu, leg. Ju-Wen Wu; 7♀, Henan: Fangchen, Dalai, VIII. 1983, ex. pupae of *Dendrolimus tabulaeformis* Tsai *et* Liu, leg. De-Long Shui; 1♂, 11♀, Anhui: Dongzhi, 1983, ex. pupae of *Dendrolimus*, leg. Ding-Xi Liao; 2♂, 3♀, Anhui: Dongzhi, 7.V.1983, ex. *Dendrolimus punctatus* Walker, leg. Ding-Xi Liao; 3♀, Anhui: Qianshan, Tianzhu Mountain, 4.IX.1976, ex. pupae of *Dendrolimus*, leg. Tao-Qian Hou; 1♀, Hubei: Wuhan, 25.VI.1980, ex. *Dendrolimus punctatus* (Walker), leg. Tao-Qian Hou; 4♀, Hunan, 25.X.1979, ex. pupae of *Pieris brassicae* L., leg. Ding-Xi Liao; 1♂, 7♀, Hunan: Chengbu, 16. VIII.1986, ex. pupae of *Dendrolimus kikuchii* Matsumura, leg. Zheng-Mao Li; 10♂, 15♀, Hunan: Daoxian, 29.XII.1979, ex. eggs of *Lebeda nobilis* Walker, leg. Ding-Xi Liao; 1♂, 5♀, Hunan: Daoxian, 12.XI.1973, ex. eggs of *Lebeda nobilis* Walker, leg. Xin-Wang Tong; 2♀, Guangxi: Nanning, VI.1975, ex. Eggs of *Dendrolimus*, leg. Lin Wei; 2♀, Guizhou: Anshun, 25.X.1980, ex. pupae of *Dendrolimus houi* Lajonquiere, leg. Jin-Rong Zhou; 6♀, Yunnan: Baoshan, 21.v.1975, ex. *Dendrolimus*, leg. Ding-xi Liao; 1♀, Tibet: Mêdog, 1100m, 26.I.1983, leg. Yin-Heng Han.

##### Hosts.

Kamijo (1982) reported *Dendrolimus spectabilis* Butler (Lepidoptera: Lasiocampidae) as a host and here we report *Dendrolimus tabulaeformis* Tsai *et* Liu, pupae/eggs of *Dendrolimus*, *Dendrolimus punctatus* (Walker), *Dendrolimus kikuchii* Matsumura, *Dendrolimus houi* Lajonquiere, *Lebeda nobilis* Walker (Lepidoptera: Lasiocampidae) and *Pieris brassicae* L. (Lepidoptera: Pieridae).

##### Distribution.

China (Beijing, Anhui, Jiangxi, Henan, Hunan, Guangxi, Guizhou, Yunnan, Tibet); Japan.

#### Dibrachys koraiensis

Taxon classificationAnimaliaHymenopteraPteromalidae

Yang, 1996

Dibrachys koraiensis Yang, 1996: 197–199, 323.

##### Diagnosis.

Body length 2.5–2.7 mm; gaster long ovate. Head in frontal view 1.25× as wide as high; antennal scrobe extending upwards and reaching anterior ocellus; lower face slightly convex; antennal insertion placed on lower ocular line; clypeus with longitudinal striation and lower margin slightly protruded, emarginate, and with two blunt teeth; lower angle of gena not exceeding clypeal margin. Antennal scape slightly shorter than eye height (0.87×); length of pedicel and flagellum shorter than head width (0.8×); anelli transverse; Fu_1_ and Fu_2_ slightly longer than broad, Fu_3_ and Fu_4_ quadrate, Fu_4_ and Fu_6_ slightly transverse. Head in dorsal view 1.88× as wide as long; eye length 2× temple length; POL 1.5× OOL. Mesosoma 1.7× as long as broad, mid lobe of mesoscutum with relatively coarse sculpture. Propodeum with complete plica and indistinct median carina. Fore wing length 2.2× width; submarginal vein 2× as long as marginal vein; marginal vein 2.53× as long as postmarginal vein, 2.13× as long as stigma vein; stigmal vein slightly longer than postmarginal vein (1.15×). Gaster 1.5× as long as broad, distinctly broader than thorax width (1.21×).

##### Material examined.

China: 1♀, Heilongjiang: Yichun, 3.VII.1972, leg. Ding-xi Liao; 1♀, Heilongjiang: Hailin, VI.1975, leg. Gui-you Zhang.

##### Hosts.

Yang (1996) reported this species as reared from the pupae of some chalcid collected from tunnels in *Picea koraiensis* Nakai (Pinales: Pinaceae) built by the wood pest *Orthotomicus golovjankoi* Pjatnitzky (Coleoptera: Curculionidae), the possible host.

##### Distribution.

China (Heilongjiang).

#### Dibrachys kunmingica

Taxon classificationAnimaliaHymenopteraPteromalidae

Jiao & Xiao sp. n.

http://zoobank.org/DC6570D7-7010-4813-89D4-570403E3A730

Figs 10–14

##### Diagnosis.

The species belongs to subgenus Allodibrachys, and similar to *Dibrachys yunnanensis* Yang has the lower angle of the gena not exceeding the clypeal margin, and the stigmal vein slightly shorter than the postmarginal vein. The main differences are: marginal vein 2× as long as stigmal vein; propodeum with plica indistinct, only conspicuous basally; median carina absent.

##### Description.

**Figures 10–18.**
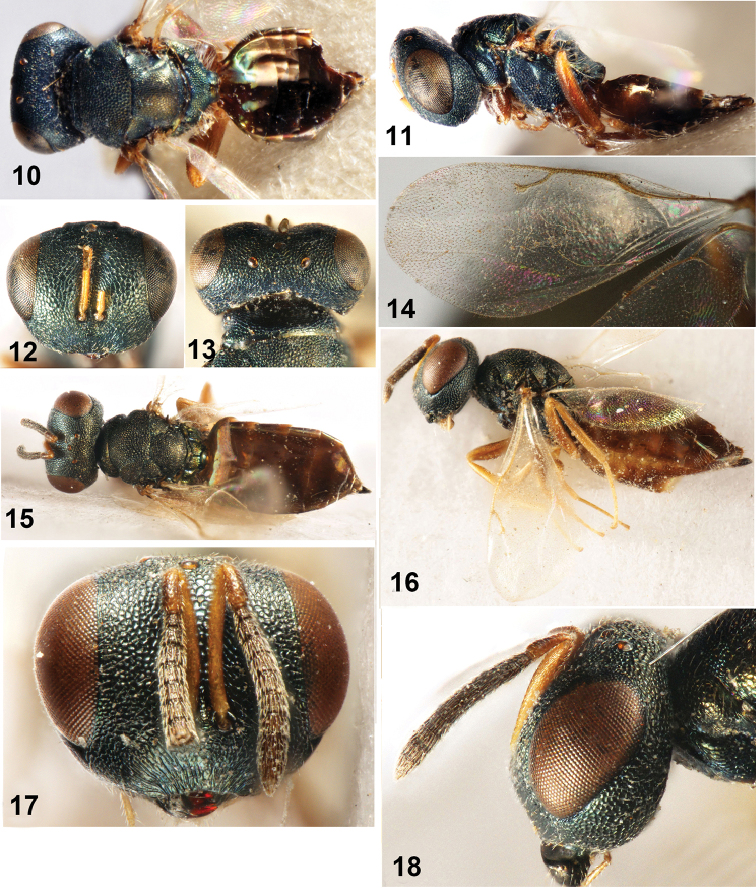
**10–14** *Dibrachys kunmingica* sp. n., female holotype **10** Body in dorsal view **11** Body in lateral view **12** Head in frontal view **13** Head in dorsal view **14** Fore wing **15–18** *Dibrachys braconidis* (Ferrière et Faure, 1925). **15** Body in dorsal view **16** Body in lateral view **17** Head in frontal view **18** Head in lateral view.

Holotype. *Female*. Body (Figs 10, 11) length 2.5 mm. Head and mesosoma dark green, with metallic reflection; gaster brown with metallic reflection basally. Antenna brown except pedicel and scape yellowish brown; legs light brown except coxae brown; fore wing hyaline, slightly infumate, wing venation yellowish-brown.

Head in frontal view (Fig. 12), width 1.29× height; frons with irregular reticulation, lower face curved ventrally; eye height 0.68× head height, inner margin of eyes slightly converging upwards, separated by 1.24× their height; antennal scrope deep, not reaching anterior ocellus; reticulation in antennal scrobe smaller than that on parascrobe. Antennal insertion slightly above lower ocular line, distance from upper margin of antennal torulus to lower margin of anterior ocellus 2× distance from lower margin of antennal torulus to clypeal margin; clypeus with dense longitudinal striation; clypeal margin slightly protruded, emarginate in the middle and with two blunt teeth, median margin concave, as a small, smooth, triangular depression; gena plump, oral fossa 0.48× as wide as head. Head in lateral view with malar sulcus inconspicuous, eye height 2.2× malar space. Antennal scape length 0.83× eye height, not reaching anterior ocellus; pedicel in lateral view 2.5× as long as broad; both anelli transverse. Head in dorsal view (Fig. 13), head 2× as wide as long; vertex convex, with regular reticulation denser than that on frons, posterior part sharply sloped down; eye length 2× temple length; POL 1.6× OOL.

Head 1.19× as broad as thorax. Mesosoma 1.37× as long as broad. Propodeum with short collar, collar subhorizontal and not margined, posterior margin smooth. Mesoscutum 2× as broad as long, reticulation on posterior area bigger than that on anterior area. Scutellum slightly convex medially, width 1.22× length, frenal line absent; reticulation shallower than that on mesoscutum posteriorly. Median length of propodeum half that of scutellum; median area flat, with deep, fine, dense reticulation; median carina absent; plica incomplete, visible anteriorly; plicae separated by 1.68× median length of propodeum; short nucha hemispheric and smooth; spiracles elongate, 2× as long as broad, separated from hind margin of metanotum by width of spiracle. Fore wing (Fig. 14) 2.36× as long as broad; without fringe from postmarginal vein to distal margin; hind wing with marginal fringe; basal vein and basal cell bare, speculum only extending to base of marginal vein; upper surface of costal cell bare, lower surface with one compact row of setae and distal 1/3 with one row of short setae and some scattered setae; submarginal vein 2× as long as marginal vein; marginal vein 1.91× as long as postmarginal vein, 2× as long as stigma vein; stigmal vein slightly shorter than postmarginal vein (0.96×); stigmal vein curved.

Petiole quadrate, as long as broad. Gaster (Fig. 10) ovate, 1.2× as long as broad, width 1.06× thorax width, length 0.94× mesosoma length; Gt_1_ covering 0.42× length of gaster, posterior margin of Gt_1_ cambered, without distinct fovea in the middle; following tergites with posterior margin straight; tergites coriaceous.

##### Material examined.

Holotype. ♀, China: Yunnan: Kunming, 25.94°N, 102.42°E, IV.1954, leg. Ding-Xi Liao. Paratype. 1♀, same data as holotype.

##### Etymology.

Named after the location of the type material.

##### Hosts.

Unknown.

##### Distribution.

China (Yunnan).

#### Dibrachys yunnanensis

Taxon classificationAnimaliaHymenopteraPteromalidae

Yang, 1996

Dibrachys yunnanensis Yang, 1996: 199–201, 324.

##### Diagnosis.

Body squat, length 2.0–2.4 mm. Head in front view 1.3× as wide as high; antennal scrobe shallow, extending upwards and reaching anterior ocellus; antennal insertion placed on the lower ocular line; clypeus with longitudinal sculpture; lower margin of clypeus slightly protruded, median emarginate and with 2 sinuate teeth; gena evenly curved, lower angle of gena not exceeding clypeal margin. Antennal scape slightly shorter than eye height, length of flagellum and pedicel combined less than head width (0.74×); pedicel in lateral view 2.2× as long as broad; both anelli transverse; Fu_1_ and Fu_5_ quadrate, Fu_6_ distinctly transverse. Head in dorsal view, 1.9× as wide as long, eye length 1.79× temple length; POL 1.78× OOL. Mesosoma 1.5× as long as broad, mid lobe of mesoscutum with regular sculpture. Propodeum with plicae complete, median carina distinct on base part. Fore wing with submarginal vein more than 2× as long as marginal vein, marginal vein 1.5× as long as postmarginal vein, stigmal vein as long as postmarginal vein. Gaster 1.3× as long as broad, as broad as thorax width.

##### Material examined.

China: 1♀, Yunnan: Nanjian, 2.VI.1980, leg. Ding-Xi Liao.

##### Hosts.

This species parasitized on larvae and pupae of *Tomicus piniperda* L. (Coleoptera: Curculionidae) which harmful to *Pinus yunnanensis* (Pinales: Pinaceae) (Yang 1996).

##### Distribution.

China (Yunnan).

#### Dibrachys (Dibrachys)

Taxon classificationAnimaliaHymenopteraPteromalidae

Förster

##### Diagnosis.

Both mandibles with four teeth; occipital carina curving, closer to vertex than foramen; fore wing without marginal fringe; gaster spindle-shaped, distinctly longer than broad.

#### Dibrachys braconidis

Taxon classificationAnimaliaHymenopteraPteromalidae

(Ferrière & Faure, 1925), new record to China

Figs 15–18

Homoporus luniger braconidis Ferrière & Faure, 1925, 11: 226.
Dibrachys braconidis (Ferrière & Faure): Bouček 1965: 30; Viggiani 1968: 112–115.

##### Diagnosis.

Body slender, length 2.0–2.9 mm (Figs 15, 16). Head in frontal view, 1.3× as wide as high; antennal scrobe extending to anterior ocellus; antennal insertion slightly above lower ocular line; lower face at least slightly convex; clypeus with longitudinal striation, lower margin of clypeus not protruding (Figs 17, 18), without tooth. Antennal scape slightly shorter than eye height, length of pedicel and flagellum shorter than head width (0.8×); Fu_1_ to Fu_3_ slightly longer than broad, Fu_4_ to Fu_6_ quadrate. Head in dorsal view, 1.9× as wide as long; eye length 2× temple length; POL 1.44× OOL. Mesosoma 1.37× as long as broad. Propodeum with complete median carina and plicae. Fore wing 2.33× as long as broad; submarginal vein 2.24× as long as marginal vein; marginal vein 1.9× as long as postmarginal vein; stigmal vein as long as postmarginal vein. Gaster 1.37× as long as broad, 1.27× as broad as thorax width.

##### Material examined.

China: 30♂, 29♀, Sichuan: Xichang, V.1992, ex. *Neodiprion xiangyunicus* Xiao *et* Zhou, leg. Zhen Zhang; 1♀, Yunnan: Nanjian, 2.VI.1980; Yunnan: Kunming, 13.VII.1977, ex. pupae of Diprioninae, leg. Jing-liang Qi; 2♀, Yunnan: Kunming, XII.1988, leg. Hong-ming Yang; 1♀, Tibet: Chamdo, 3400m, 15.VIII.2001, leg. Chao-dong Zhu.

##### Hosts.

The species mainly parasitizes *Luffia ferchaultella*, and *Luffia lapidella* (Lepidoptera: Psychidae) and *Apanteles glomeratus* (L.) (Hymenoptera: Braconidae) (Graham, 1969). Here we newly report *Neodiprion xianyunicus* Xiao *et* Zhou and Diprionidae sp. (Hymenoptera: Symphyta).

##### Distribution.

China (Sichuan, Yunnan, Tibet); Palearctic and Nearctic regions. This is the first record from the Oriental region.

#### Dibrachys confusus

Taxon classificationAnimaliaHymenopteraPteromalidae

(Girault, 1916), new record to China

Figs 19–22

Coelopisthia confusus Girault, 1916a: 246.
Dibrachys confusus (Girault): Peck 1951: 554; Grissell 1974: 318; Burks 1979: 828.

##### Diagnosis.

**Figures 19–28.**
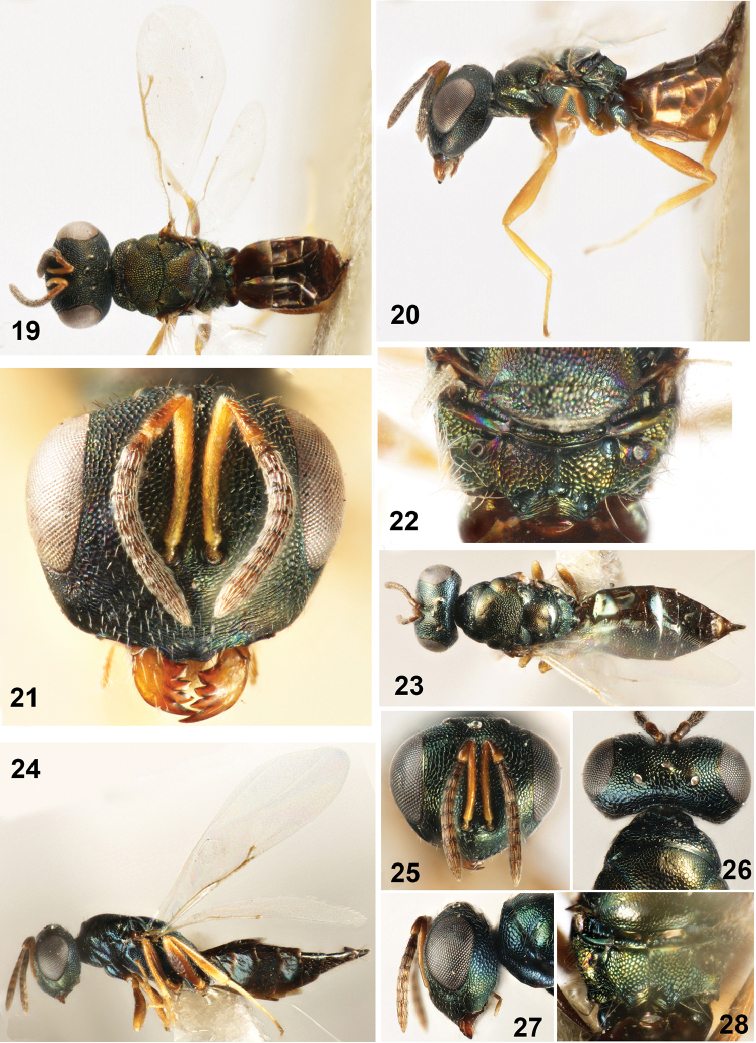
**19–22** *Dibrachys confusus* (Girault, 1916) **19** Body in dorsal view **20** Body in lateral view **21** Head in frontal view **22** Propodeum **23–28** *Dibrachys golmudica* sp. n., female holotype **23** Body in dorsal view **24** Body in lateral view **25** Head in frontal view **26** Head in dorsal view **27** Head in lateral view **28** Propodeum.

Body slightly slender (Figs 19, 20), about 2.6 mm. Head in frontal view (Fig. 21) 1.15× as wide as high; antennal scrobe extending upwards and not reaching anterior ocellus; antennal insertion slightly above lower ocular line; lower face flat; clypeus with transverse striation and lower margin protruding, emarginate with two blunt teeth. Antennal scape slightly shorter than eye height (0.91×) but reaching lower margin of anterior ocellus; length of pedicel and flagellum combined shorter than head width; anelli transverse; each funicular segment slightly longer than its broad respectively. Head in dorsal view with width 2× length; eye length 1.87× temple length; POL 1.46× OOL. Mesosoma 1.43× as long as broad, mesoscutum with regular sculpture. Propodeum with median carina complete (Fig. 22), plicae complete and parallel anteriorly. Fore wing 2.38× as long as broad ; submarginal vein 2.37× as long as marginal vein; marginal vein 1.72× as long as postmarginal vein; stigmal vein as long as postmarginal vein. Gaster 2× as long as broad, narrower than thorax width.

##### Material examined.

China: 3♀, Beijing: Yuanmingyuan Imperial Garden, 2.VI.1984, ex. larvae of *Lymantria dispar* (L.), leg. Ding-Xi Liao.

##### Hosts.

De Santis (1983) reported *Megachile rotundata* (Fabricius) (Hymenoptera: Megachilidae) as a host and here we record the larvae of *Lymantria dispar* (L.)(Lepidoptera: Lymantriidae).

##### Distribution.

China (Beijing); Palearctic, Nearctic and Neotropical regions.

#### Dibrachys golmudica

Taxon classificationAnimaliaHymenopteraPteromalidae

Jiao & Xiao sp. n.

http://zoobank.org/F39A82B6-44C1-4E9A-9D23-B4462E870754

Figs 23–28

##### Diagnosis.

The new species belongs to *Dibrachys* *s. str.*, and the noticeable differences with other species of the subgenus by the following characters: in female, antennae with three anelli; the postmarginal vein being distinctly shorter than the stigmal vein (0.5×), and gaster being 2.5× as long as broad.

##### Description.

Holotype. *Female*. Body length 2.2 mm (Figs 23, 24). Head and mesosoma black with bronze luster and metallic reflection. Gaster dark brown with metallic reflection basally. Antennal scape yellowish brown to light brown from base to apex, flagellum dark brown; legs yellowish brown except coxae concolorous with body and femora light brown; fore wing hyaline, without infumation, venation yellowish brown or yellowish.

Head in frontal view (Fig. 25) 1.24× as wide as high; eyes with inner margins parallel, eye height 0.62× head height, eyes separated by 1.26× their height; lower face with weak striation, upper face with obvious regular reticulation; antennal scrobe deep, not reaching anterior ocellus. Antennal insertion on lower ocular line, distance from upper margin of torulus to lower margin of anterior ocellus 2.54× distance from lower margin of torulus to lower margin of clypeus; clypeus with longitudinal striation on both sides, lower margin slightly protruded, emarginate in middle with two obtuse teeth; oral fossa 0.49× as wide as head. Head in lateral view (Fig. 27) with malar sulcus conspicuous, eye height 1.5× its broad and 2.83× malar space. Antennal scape length 0.91× eye height; length of flagellum and pedicel combined less than head width (0.88×); pedicel in lateral view 2.3× as long as broad; antenna with 3 anelli, Fu_1_ and Fu_2_ distinctly transverse, Fu_3_ quadrate, Fu_1_ to Fu_3_ combined 0.78× as long as pedicel; Fu_4_ longer than broad, Fu_5_ quadrate; each funicular segment with one row of sensilla; setae on funicle all decumbent; clava not distinctly clavate, 3.4× as long as broad, micropilosity limited to apex of third clava segment. Head in dorsal view (Fig. 26), 2× as wide as long; vertex convex, sculpture on vertex slightly smaller than sculpture on frons; occipital carina distinct; eye length 2.5× temple length; POL 2.11× OOL.

Head 1.24× as broad as thorax. Mesosoma 1.6× as long as broad. Pronotum 0.65× as broad as mesoscutum, collar rounded, posterior band smooth. Mesoscutum 1.57× as broad as long, with regular reticulation, in anterior half weakly reticulate and posterior half with deep reticulation; notauli distinct but not complete. Scutellum convex, 1.07× as broad as long, frenal line absent; reticulation smaller than on mesoscutum, but regular and shallow. Propodeum medially ½ as long as scutellum, with fine, deep, dense reticulation; plica weak (Fig. 28), only visible basally and separated by 2× medial length of propodeum; median carina incomplete; propodeum with short, slightly convex nucha having transverse striation; propodeal spiracles elongate, 2.67× as long as broad. Fore wing 2.16× as long as broad, without marginal fringe; setae pale, inconspicuous; basal vein and basal cell bare, upper surface of costal cell bare, lower surface with one complete row of setae and distally with some scattered setae; submarginal vein 2.75× as long as marginal vein, marginal vein 2.63× as long as stigmal vein, postmarginal vein shorter than stigmal vein (at most 0.5×); stigmal vein slightly curved.

Gaster spindle-shaped with apex pointed (Fig. 23), 2.5× as long as broad; as wide as thorax; Gt_1_ covering 1/4 of gaster, with posterior margin cambered; tergites beyond Gt_1_ equal in length; ovipositor exserted.

*Male*. Head black except frons with yellowish-green, and antennae yellow; mesosoma black except thorax purplish laterally, legs yellow except coxae brown. Antennae with two distinctly transverse anelli, pedicel in lateral view 1.8× as long as broad, each funicular segment longer than broad; gaster oval, apex not pointed.

##### Material examined.

Holotype. ♀, China: Qinghai: Golmud, Guolemude, 2880m, 36.26°N, 94.53°E, 14.IX.2001, leg. Chao-Dong Zhu. Paratype. 1♀, same data to holotype; 3♂, 6♀, Inner Mongolia: Ejin B., 11.VI.1981, ex. *Dinorhopala* on *Populus diversifolia*, leg. Hua-Qiang Shao.

##### Etymology.

Named after the location where the holotype was collection.

##### Hosts.

Specimens from Inner Mongolia were reared from *Dinorhopala* (Coleoptera: Curculionidae) on *Populus diversifolia*.

##### Distribution.

China (Inner Mongolia, Qinghai).

#### Dibrachys liaoi

Taxon classificationAnimaliaHymenopteraPteromalidae

Jiao & Xiao sp. n.

http://zoobank.org/2418A0BD-799F-44D4-8A3B-BCCAB3FCF7C2

Figs 29–32

##### Diagnosis.

The new species belongs to *Dibrachys* *s. str.*, and the mainly differences with *Dibrachys microgastri* (Bouché) as follows: *Dibrachys liaoi* sp. n. slightly blue-greenish, clypeal margin with two sharp teeth, Fu_1_ to Fu_4_ length slight longer than its width respectively, Fu_5_ and Fu_6_ quadrate, gaster 1.8× as long as broad; but in *Dibrachys microgastri* (Bouché), body yellow-green, clypeal margin with two blunt teeth, Fu_1_ to Fu_5_ quadrate, Fu_6_ transverse, gaster 2× as long as broad.

##### Description.

**Figures 29–37.**
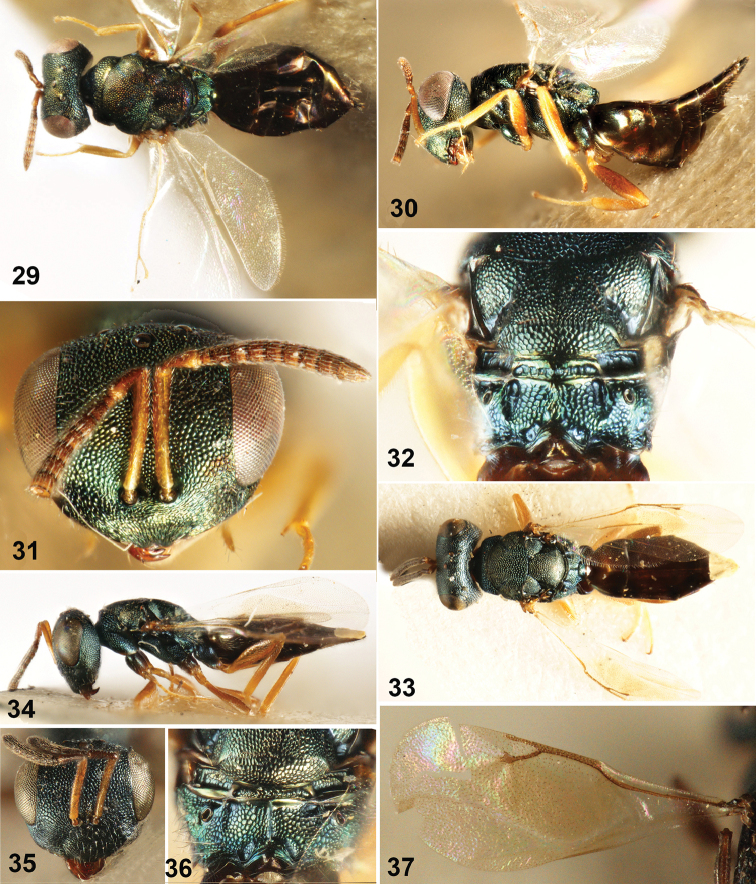
**29–32** *Dibrachys liaoi* sp. n., female holotype **29** Body in dorsal view **30** Body in lateral view **31** Head in frontal view **32** Propodeum **33–37** *Dibrachys maculipennis* Szelényi **33** Body in dorsal view **34** Body in lateral view **35** Head in frontal view **36** Propodeum **37** Fore wing.

Holotype. *Female*. Body (Figs 29, 30) length 2.2 mm. Head and mesosoma dark green, with metallic reflection; gaster brown and with metallic reflection basally. Antennae dark brown except scape and pedicel yellowish brown; mandible brown; legs yellowish brown except coxae brown; fore wing slightly infumate, wing venation yellowish brown.

Head in frontal view (Fig. 31), width 1.24× height; frons with dense reticulation; lower face flat, reticulation on lower face same as that on frons; eye height 0.7× head height, eyes separated by 1.09× eye height; antennal scrobe deep, extending upwards but not reaching anterior ocellus. Antennal insertion on lower ocular line, distance from upper margin of torulus to lower margin of anterior ocellus 2.35× distance from lower margin of torulus to clypeal margin; clypeus with longitudinal sculpture, only small area smooth; clypeal margin protruded, emarginate in middle with two sharp teeth; oral fossa width 0.46× head width. Head in lateral view, malar sulcus inconspicuous, eye height 3.3× malar space. Antennal scape 0.81× as long as eye height, not reaching lower margin of anterior ocellus; length of pedicel and flagellum combined shorter than head width (0.84×); pedicel in lateral view 2.6× as long as broad; anelli transverse; Fu_1_ to Fu_4_ slightly longer than broad respectively, Fu_5_ and Fu_6_ quadrate; each funicular segment with one row of longitudinal sensilla; clava slightly clavate, 2.43× as long as broad, micropilosity only limited to apex of third clava segment. Head in dorsal view 1.89× as wide as long; vertex convex, occipital carina strong; eye length 2× temple length; POL 1.64× OOL.

Head 1.31× as broad as thorax. Mesosoma 1.38 × as long as broad. Pronotum with raised reticulation, pronotal collar slightly narrower than mesoscutum (0.86×); middle length of pronotum almost 1/9 as long as length of mesoscutum; collar not margined anteriorly, posterior margin of collar with a smooth band. Mesoscutum 1.86× as broad as long, with regular and dense reticulation; notauli incomplete and unconspicuous. Scutellum convex, 1.09× as broad as long, frenal line absent; reticulation same as on mesoscutum but slightly large on posterior part of scutellum. Propodeum (Fig. 32) medially ½ as long as scutellum; plica complete; median carina incomplete, occasionally with one or two short longitudinal ridge which interrupted in the middle; nucha short and smooth, separated with middle part of propodeum by a transverse shallow depression; spiracles elongate, 2× as long as broad, separated by the width of spiracles from hind margin of metanotum; area below spiracles with finely reticulation. Fore wing 2.25× as long as broad, without marginal fringe; basal vein with sparse setae, basal cell bare; speculum only stretched to 1/3 base of marginal vein; upper surface of costal cell bare, lower surface with a one complete row of setae and distal 1/3 with some scattered setae; submarginal vein 2.33× as long as marginal vein; marginal vein 1.67× as long as postmarginal vein; stigmal vein as long as postmarginal vein, slightly curved.

Petiole invisible dorsally. Gaster (Fig. 29) long ovate, 1.8× as long as broad, 1.3× as broad as thorax width; surface of each tergite coriaceous; Gt_1_ covering 1/3 length of gaster, posterior margin of Gt_1_ cambered, median with an obvious hollow; following tergites with posterior margin straight; gaster terminal acute.

Male. Body length 2.1 mm; head and thorax blue-green; antenna light brownish except clava slightly dark, other segments yellow; legs yellow to yellowish brown except coxae concolorous with body; fore wing yellowish brown; gaster brown, with a yellow transverse bright ribbon at 1/3 base of gaster.

##### Material examined.

Holotype: China: ♀, Beijing: Miyun Reservoir, 40.29°N, 116.50°E, 18.VII.1983, ex. pupae of *Dendrolimus tabulaeformis*, leg. Ju-Wen Wu. Paratypes: China: 1♂, 2♀, same data as holotype; 2♀, Beijing: Miyun Reservoir, 20.VII.1983, ex. pupae of *Dendrolimus tabulaeformis*, leg. Ju-Wen Wu; 5♀, Beijing: Huairou, 15.VI.1982, ex. *Illiberis pruni*, leg. Mr. Jin; 1♀, Beijing: Songshan, 26.VIII.1984, ex. *Illiberis pruni*; 4♀, Beijing: Changping, 15.VI.1981, ex. *Locastra muscosalis*, leg. Zhen-Hua Liu; 1♀, Beijing: Yuanmingyuan Imperial Garden, 18.VII.1984, ex. larvae of *Lymantria dispar*, leg. Mu-Zong Cheng; 6♀, Beijing: Yuanmingyuan Imperial Garden, 2.VI.1984, ex. larvae of *Lymantria dispar*, leg. Ding-Xi Liao; 4♂, 5♀, Beijing: Mentougou, late July of 1983, ex. *Prothesia similes xanthocampa*, leg. Sui-Hua Zhao; 1♀, Beijing: Qingbaichang, leg. Ding-Xi Liao.

##### Etymology.

In memory of professor Ding-xi Liao in China.

##### Hosts.

Larvae of *Illiberis pruni* Dyar, *Illiberis nigra* Leech (Lepidoptera: Zygaenidae), *Lymantria dispar* (L.) (Lepidoptera: Noctuoidea) and *Porthesia similis* (Fueszly) (Lepidoptera: Lymantridae), pupae of *Dendrolimus tabulaeformis* Tsai *et* Liu, *Dendrolimus superans* (Butler) (Lepidoptera: Lasiocampidae), *Illiberis ulmivora* Graeser, *Pseudopanolis flavimacula* Inaba (Lepidoptera: Noctuidae), *Rogas dendrolimi* (Matsumura) (Hymenoptera: Braconidae) and Tenthredinidae sp..

##### Distribution.

China (Liaoning, Beijing, Hebei, Shanxi, Shandong, Gansu, Qinghai).

#### Dibrachys maculipennis

Taxon classificationAnimaliaHymenopteraPteromalidae

Szelényi, 1957, new record to China

Figs 33–37

Dibrachys maculipennis Szelényi, 1957: 301, 307.

##### Diagnosis.

Body slender (Figs 33, 34), length 2.2–2.3 mm; gaster spindle. Head in frontal view (Fig. 35), 1.13× as wide as high; antennal scrobe very shallow, extending upwards but not reaching anterior ocellus; antennal insertion slightly above lower ocular line; lower face flat; clypeus with transverse striation and lower margin slightly protruding with two blunt teeth. Antennal scape as long as eye height, reaching lower margin of anterior ocellus; length of pedicel and flagellum combined slightly shorter than head width (0.95×); anelli transverse; Fu_1_ to Fu_3_ slightly long than broad respectively, Fu_4_ and Fu_5_ quadrate, Fu_6_ slightly transverse; clava slightly clavate, 2× as long as broad. Head in dorsal view, 2× as wide as long; occipital carina strong; POL 1.5× OOL. Mesosoma 1.6× as long as broad, with regular reticulation. Propodeum (Fig. 36) with median carina incomplete; plicae distinct anteriorly. Fore wing (Fig. 37) with a yellowish-brown infumation behind marginal vein; submarginal vein 2× as long as marginal vein, marginal vein 1.94× as long as postmarginal vein, 1.5× as long as stigmal vein; stigma vein slightly longer than postmarginal vein (1.1×). Gaster 2× as long as broad, 1.14× as broad as thorax width.

##### Material examined.

China: 2♀, Beijing, V1967; 1♀, Shaanxi: Hangzhou, X.1959, ex. *Smerinthus planus* Walker, leg. Zhe-Min Zheng; 1♀, Zhejiang: Hangzhou, 7.VI.1972, ex. *Apanteles baoris* Wilkinson, leg. Ding-Xi Liao.

##### Hosts.

Recorded hosts of the species were *Megachile rotundata* (Fabricius) (Hymenoptera: Megachilidae) (Peck 1969) and *Hyphantria cunea* (Drury) (Lepidoptera: Erebidae) (Dzhanokmen 1978). Here we newly report *Smerinthus planus* Walker (Lepidoptera: Sphingidae) and *Apanteles baoris* Wilkinson (Hymenopter: Braconidae).

##### Distribution.

China (Beijing, Shaanxi, Zhejiang); Palearctic, Nearctic and Neotropic regions.

#### Dibrachys microgastri

Taxon classificationAnimaliaHymenopteraPteromalidae

(Bouché, 1834)

Figs 38–41

Diplolepis microgastri Bouché, 1834: 168; neotype female in ZMH by Peters and Baur 2011: 12. Syntypes presumed lost (Graham 1969: 811). Möller 1886: 83; Vidal 2001, 7: 62.
Dibrachys microgastri (Bouché) Peters & Baur, 2011: 18; Vidal 2001: 62.
Dibrachys microgastri Synonymy: Pteromalus cavus Walker, 1835: 477–478; Pteromalus decedens Walker, 1835: 478; Pteromalus albinervis Ratzeburg, 1844: 199; Pteromalus boucheanus Ratzeburg, 1844: 196; Pteromalus tenuis Ratzeburg, 1844: 195; Pteromalus zelleri Ratzeburg, 1848: 190; Pteromalus vesparum Ratzeburg, 1852: 233; Cleonymus clisiocampae Fitch, 1856: 431–432; Pteromalus boarmiae Walker in Newman, 1863: 8609, 8610; Cheiropachus nigro-cyaneus Norton, 1869: 327; Eupelmus cereanus Rondani, 1876: 38. 40; Pteromalus gelechiae Webster, 1883: 151; Pteromalus chionobae Howard, 1889: 1872, 1889; Arthrolytus apatelae Ashmead, 1893: 162; Arthrolytus pimplae Ashmead, 1894: 339; Trichomalus truyilloi Blanchard, 1938: 178; Tritneptis elegans Szelényi, 1981: 400.

##### Diagnosis.

**Figures 38–47.**
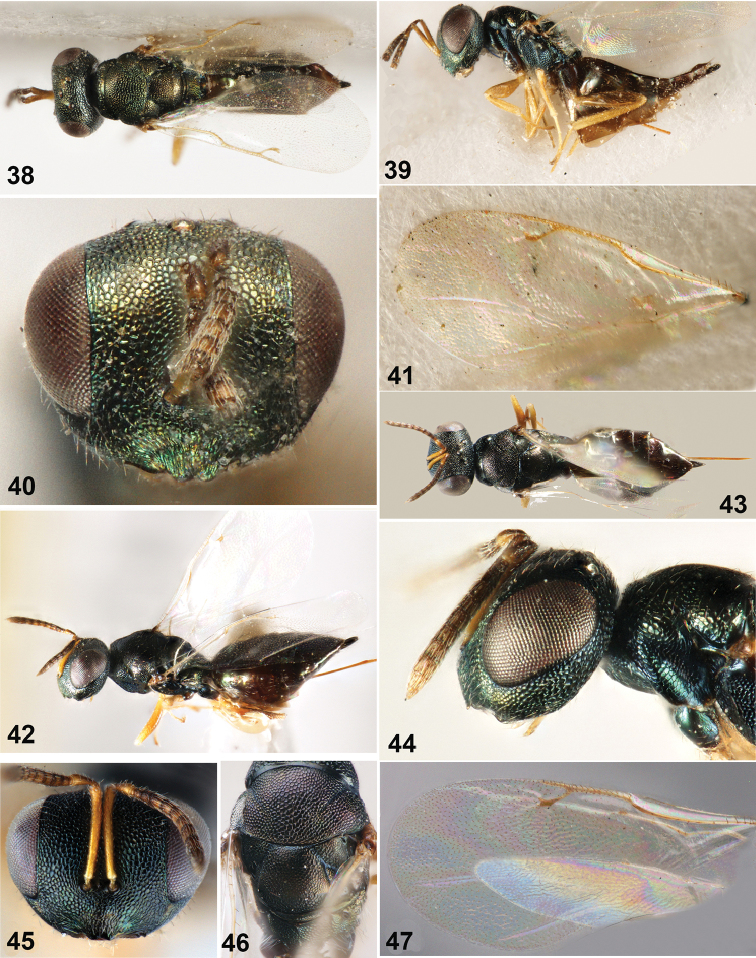
**38–41** *Dibrachys microgastri* (Bouché) **38** Body in dorsal view **39** Body in lateral view **40** Head in frontal view **41** Fore wing **42–47** *Dibrachys qinghaiensis* sp. n., female holotype **42** Body in lateral view **43** Body in dorsal view **44** Head in lateral view **45** Head in frontal view **46** Mesoscutum **47** Fore wing.

Body slender (Figs 38, 39), length 1.8–2.5 mm; gaster long ovate, spindle-shaped. Head in frontal view (Fig. 40), width 1.21× height; antennal scrobe extending upwards but not reaching anterior ocellus; antennal insertion placed on lower ocular line; lower face at least slightly convex; sculpture on face larger than on vertex; clypeus with longitudinal striation; lower margin of clypeus slightly protruded, emarginated and with two blunt teeth. Antennal scape slightly shorter than eye height; not reaching lower margin of anterior ocellus; length of pedicle and flagellum shorter than head width; anelli transverse; each funicular segment quadrate except Fu_6_ transverse; setae on antenna become an angle with antennal surface. Mesosoma 1.43× as long as broad; mid lobe of mesoscutum with coarse sculpture. Propodeum with complete plicae and weak median carina. Fore wing (Fig. 41) 1.88× as long as broad; submarginal vein 2× as long as marginal vein, marginal vein 1.9-2.5× postmarginal vein, postmarginal vein shorter or as most as long as stigmal vein; stigmal vein straight. Gaster 2× as long as broad, slightly broader than thorax width.

##### Material examined.

China: 3♂, 7♀, Heilongjiang: Hailin, VI.1975, ex. pupae of *Atractodes* sp., leg. Gui-You Zhang; 2♂, 1♀, Heilongjiang: Yichun, 3.VII.1972, ex. Tachinidae sp. on Tortricidae sp., leg. Ding-Xi Liao; 1♂, 8♀Heilongjiang: Yichun, 13.VII.1962, ex. Tachinidae sp. on *Ptycholomoides aeriferanus* Herrich-Schaffer, leg. Ding-Xi Liao; 2♂, 6♀, Heilongjiang: Yichun, 18.IX.1975, ex. *Ptycholomoides aeriferanus* Herrich-Schaffer, leg. You-Qiao Liu; 4♀, Heilongjiang: Harbin, 3.VII.1962, ex. pupae of *Yponomeuta padella* Linnaeus, leg. Tai-Lu Chen; 5♀, Heilongjiang: Harbin, 4.VII.1962, ex. pupae of flies, leg. Tai-Lu Chen; 1♂, 2♀, Heilongjiang: Harbin, 11.V.1978, ex. pupae of *Anacampsis populella* Clerck, leg. Wen-Min Chen; 2♂, 7♀, Heilongjiang: Harbin, 11.IV.1978, ex. larvae of Aphididae sp., leg. Wen-Min Chen; 11♂, Heilongjiang: Jiamusi, 21.IX.1979, ex. aphids on cabbagecabbage aphid, leg. Ding-Xi Liao; 1♂, 8♀,Heilongjiang: Mishan, VI.1963, ex. *Pyrausta nubilalis* (Hübner), leg. De-Yun Deng; 1♂, 1♀, Heilongjiang: Dailing, 18.IX.1975, ex. *Rhyacionia buoliana*, leg. Ding-Xi Liao; 2♀, Jilin: Siping, VII.1980, ex. *Coleophoridae* sp., leg. Yu-Ying Qiu; 5♀, Liaoning: Liaoyang, 19.VI.1980, ex. larvae of *Lymantria dispar* L., leg. Yu-Bao Zhang and Gui-Zhi Zhang; 3♂, 7♀, Liaoning: Liaoyang, 17.VII.1979, ex. *Musca domestica* (Linnaeus,1758), leg. Ding-Xi Liao; 1♀, Liaoning: Liaoyang, 10.VI.1979, ex. pupae of *Cnidocampa flavescens* (Walker), leg. Yu-Bao Zhang; 5♀, Liaoning: Jinzhou, vi.1970, ex. *Nephoteryx pirivorella* Matsumura, leg. Bin Liu; 1♂, 1♀, Liaoning: Shenyang, 11.VII.1978, ex. sawfly, leg. Gong-Tian Xu; 1♂, 1♀, Liaoning: Xingcheng, 25.V.1981, leg. Yan-Li Zhao; 4♀, Liaoning: Suizhong, 13.IX.1973, ex. pupae of Tachinidae sp., leg. Shu-Hai Wang; 5♀, Liaoning: Liaoyang, Yuejiadadui, 1979, ex. pupae of *Lymantria dispar* (L.) from *Populus* sp., leg. Yu-bao Zhang & Gui-Zhi Zhang; 1♀, Liaoning: Liaoyang, Beiling, 5.IX.1978, ex. *Lymantria dispar* (L.), leg. Gong-Tian Xu; 1♀, Liaoning: Fuxian, 25.VI.1976, ex. Tortricidae sp., leg. Ding-Xi Liao; 3♀, Liaoning: Siping, VI.1989, ex. Yponomeutidae sp., leg. Gui-You Zhang; 8♀, Liaoning: Fusong, IX.1953, ex. eggs of *Dendrolimus* sp.; 2♀, Inner Mongolia: Urad Middle Banner, 17.VII.1980, ex. *Anacampsis Populella* Clerck, leg. Xu-Chang Huang; 3♂, Inner Mongolia: Horinger, 4.VIII.1981, ex. *Malacosoma* sp., leg. Qiang-Hua Shao; 1♂, 2♀, Inner Mongolia: Baotou, 19.IX.1989, ex. pupae of gelechiid moth, leg. Zhong-Ren Liu; 2♂, 3♀, Inner Mongolia: Baotou, 24.VI.1989, ex. pupae of ichneumon, leg. Zhong-Ren Liu; 1♀, Inner Mongolia: Baotou, 28.vii.1985, ex. Scolytidae sp., Zhong-Ren Liu; 3♂, 7♀, Beijing, vii.1986, ex. pupae of Tachinidae sp., leg. Da-Wei Huang; 1♂, 9♀, Beijing, 21.VIII.1962, ex. Tortricid, leg. Ding-Xi Liao; 5♀, Beijing: Xijiao, 20.IX.1957, ex. pupae of *Stilprotia salicis*, leg. Tai-Lu Chen; 1♀, Beijing: Miyun, 17.VI.1984, ex. *Dendrolimus tabulaeformis*, leg. Da-Wei Huang; 2♂, 6♀, Beijing: Daxing, 1963, ex. obsolete honeycomb, leg. Zong-You Xu; 2♀, Beijing: Yuanmingyuan Imperial Garden, 25.vii.1984, ex. larvae of *Lymantria dispar*, leg. Mu-Zong Cheng; 4♀, Hebei: Zhangbei, VII-XII.1983, ex. *Stilpnotia candida*, leg. Ding-Xi Liao; 8♂, 24♀, Hebei: Zhangbei, 21.VII.1983, larvae of *Stilpnotia candida*, leg. Xing-Jun Li; 9♀, Hebei: Zhangbei, vii.1983, ex. Lymantridae sp., leg. Xing-Jun Li; 1♂, 5♀, Hebei: Zhangbei, ex. pupae of *Gypsonoma minutara*, leg. Jun-Rong Dai; 1♀, Hebei: Fengning, 20.v.1992, ex. *Rogas dendrolimi* (Matsumura), leg. Da-Zhou Wang; 2♂, 3♀, Shanxi: Taiyuan, 6.V.1991, ex. *Eulecanium gigantean*, leg. Hui-Di Zhang; 2♀, Shanxi: Taigu, 8.VII.1979, ex. pupae of Yponomeutidae sp., leg. Zhan-Gui Li; 1♂, 2♀, Shanxi: Taiyuan, 19.vi.1990, ex. pupae of *Yponomeuta polystinellus* Felder, leg. Da-Wei Huang; 1♂, 1♀, Shanxi: Taiyuan, iv.1980, ex. pupae of *Ancylis sativa* Liu, leg. Ci Yu; 3♀, Shanxi: Pingshun, 10.VIII.1978, ex. *Pinus tabuliformis* Carrière; 1♂, 1♀, Shanxi: Taigu, 8.VII.1979, ex. pupae of *Galleria mellonella*, leg. Zhan-Gui Li; 3♀, Shanxi: Taigu, 15.VI.1979, ex. larvae of *Illiberis nigra* Leech, leg. Zhan-Gui Li; 4♀, Shanxi: Taigu, VII.1979, ex. *Illiberis nigra* Leech, leg. Zhan-Gui Li; 1♀, Shanxi: Taigu, VII.1979, ex. *Lithocolletis ringoniella* Mats., leg. Zhan-Gui Li; 2♀, Shanxi: Shuoxian, 24.V.1984, ex. Braconidae on *Coccinella septempunctata*, leg.Yu-Zhi Niu; 3♂, 6♀, Shanxi: Shuoxian, 3-16.VI.1984, ex. Braconidae sp. on *Anacampsis Populella* Clerck, leg.Yu-Zhi Niu; 3♂, 6♀, Shanxi: Shuoxian, 14-18.VI.1984, ex. *Anacampsis Populella* Clerck, leg. Yu-Zhi Niu; 1♂, 2♀, Shanxi: Shuoxian, 20.VI.1983, ex. *Anacampsis Populella* Clerck, leg.Yu-Zhi Niu; 1♂, 7♀, Shanxi: Shuoxian, V-VII.1984, ex. Tachinidae sp. on *Anacampsis Populella* Clerck, leg.Yu-Zhi Niu; 1♀, Shangdong: Weihai, 3.VI.1958, leg. Jin-Long Mao; 1♀, Shangdong: Fushan, 26.X.1958, leg. Jin-Long Mao; 1♂, 4♀, Henan: Anyang, 20.V.1956, ex. *Pectinophora gossypiella* (Saunders), leg. Ding-Xi Liao; 3♀, Henan: Zhengzhou, 10.IX.1972, ex. larvae of Olethreutidae sp., leg. Ding-Xi Liao; 1♀, Shaanxi: Xianyang, 6.III.1975, ex. pupae of *Earias cupreoviridis* Walker, leg. Ding-Xi Liao; 2♂, 6♀, Ningxia: Luhuatai, 29.V.1982, ex. *Ypsolopha vittellus* (Linnaeus), leg. Ding-Xi Liao; 3♀, Ningxia: ZhongweisShapotou, 15.VI.1981, leg. Ding-Xi Liao; 3♀, Ningxia: Yinchuang, 12.VI.1974, ex. pupae of Syrphidae sp. on *Ulmus pumila* L.; 1♂, 9♀, Gansu: Pingliang, VII.1966, ex. Tachinidae sp. & *Illiberis pruni*, leg. Shou-Min Liu; 3♀12♂, Gansu: Pingliang, VII. 1966, ex. *Illiberis pruni*, leg. Shou-Min Liu; 4♂, 22♀, Xinjiang: Altay, VI.1979, ex. larvae of *Gelechia pinguinella* Trietschke, leg. Jun-Wen Xia; 5♀, Xinjiang: Korla, collecting time unknown, ex. eggs of *Macroglossum corythus luteata* (Butler), leg. Tai-Lu Chen; 3♀, Xinjiang: Urumchi, 12.VII.1980, ex. pest on *Salix* sp., leg. Jiu-Xiong Bai; 1♂, 5♀, Jiangsu: Nanjing, 4.VII.1963, leg. Ding-Xi Liao; 2♂, 5♀, Shanghai: Pudong New District, III.1972, ex. cottonseed; 4♀, Shanghai: Minhang, XII.1979, ex. *Apanteles glomeratus* (L.), leg. Ji-Long He; 6♀, Anhui: Dangshan, 7.XI.1975, ex. *Pyrausta nubilalis* (Hubern) & *Chilo infuscatellus* (Snellen), leg. Ding-Xi Liao; 12♀, Anhui:Huangfu Mountain, 19.VI1965, ex. larvae of Lepidoptera, leg. Ding-Xi Liao; 1♂, 4♀, Zhejiang: Hangzhou, X.1954, ex. *Pectinophora gossypiella* (Saunders); 2♂, 6♀, Zhejiang: Hangzhou, i.1963, leg. Ding-Xi Liao; 5♂, 2♀, Zhejiang: Hangzhou, I.1963, ex. *Pectinophora gossypiella* (Saunders), leg. Cui Hu; 1♂, 2♀, Hubei: Hong’an, V.1978, ex. pupae of *Dendrolimus punctatus* Walker, leg. Ding-Xi Liao; 6♀, Hunan: Changsha, 20.III.1984, ex. *Chilo suppressalis* (Walker), leg. Ding-Xi Liao; 1♂, 9♀, Hunan: Liuyang, 24.IX.1979, ex. eggs of *Dendrolimus* sp. , leg. Ding-Xi Liao; 1♀, Hunan, collecting time unknown, leg. Xin-Wang Tong; 1♀, Yunnan: Kunming, 17.V.1967, ex. larvae of *Macrocentrus* sp. leg. Jing-Liang Qi; 1♀, Yunnan: Zhaotong, 5.IV.1973, ex. *Chilo suppressalis* (Walker), leg. Ding-Xi Liao; 1♂, 1♀, Tibet: Lhasa, 3650m, 27.VIII.2001, leg. Chao-Dong Zhu. 2♀, Sk. Ahus, 15.IV.1979. leg. K. J. Hedqvist, *Dibrachy cavus* (Walker), det. K. J. Hedqvist; N. Zealand: 1♀, Lincoln, nr Christchurch RT., 1988, B.J. Donovan. Cult. Vespula gemanica, *Dibrachys boarmiae* (Walker), det. Z. Boucěk, 1988.

##### Hosts.

Hosts of *Dibrachys microgastri* has been widely recorded, and the primary parasite has been recorded more than 240 species from 45 families of seven insects orders and also recorded two species of Arachnida (Noyes 2002). In our study, the species was parasitic on Coleoptera (Scolytidae sp.), Diptera (Tachinidae sp. on Tortricidae sp., Tachinidae sp. on *Anacampsis Populella* Clerck, pupae of Tachinidae sp. and Syrphidae sp. on *Ulmus pumila* L.), Hemiptera (aphids on cabbage), Hymenoptera (*Apanteles glomeratus* (L.), Braconidae sp. on *Coccinella septempunctata*, larvae of *Macrocentrus* sp., pupae of *Gelis* sp. and Lepidoptera (*Chilo infuscatellus* (Snellen), *Chilo suppressalis* (Walker), *Lymantria dispar* (L.), *Malacosoma* sp., *Nephopteryx pirivorella* (Matsumura), *Pectinophora gossypiella* (Saunders), *Ptycholomoides aeriferanus* (Herrich-Schäffer), *Pyrausta nubilalis* (Hübner), *Ypsolopha vittellus* (Linnaeus), eggs of *Dendrolimus* sp., larvae of *Gelechia pinguinella* Trietschkeb and Olethreutidae sp., pupae of *Anacampsis populella* Clerck, pupae of *Cnidocampa flavescens* (Walker), pupae of *Dendrolimus punctatus* (Walker), pupae of *Earias cupreoviridis* Walker, pupae of *Stilprotia salicis* (L.), pupae of *Yponomeuta padella* Linnaeus and so on).

##### Distribution.

China (Heilongjiang, Jilin, Liaoning, Inner Mongolia, Beijing, Hebei, Shanxi, Shangdong, Henan, Shaanxi, Ningxia, Gansu, Xinjiang, Jiangsu, Shanghai, Anhui, Zhejiang, Hubei, Hunan, Yunnan, Tibet); widespread world-wide distribution (Noyes, 2016).

#### Dibrachys qinghaiensis

Taxon classificationAnimaliaHymenopteraPteromalidae

Jiao & Xiao sp. n.

http://zoobank.org/2C61C736-41D3-4EB0-8C0B-F3218A96B622

Figs 42–47

##### Diagnosis.

The new species belongs to *Dibrachys* s. str., and the mainly differences with *Dibrachys microgastri* (Bouché) are as follows: antenna of *Dibrachys qinghaiensis* sp. n. slender, each funicular segment at least slightly longer than its broad; antennal scape as long as eye height, and nearly reaching lower margin of anterior ocellus; but *Dibrachys microgastri* (Bouché) at least with several transverse funicular segment in distal of antenna, antennal scape distinctly shorter than eye height, and not reaching lower margin of anterior ocellus.

##### Description.

Holotype. *Female*. Body (Figs 42, 43) length 2.2 mm. Head and mesosoma dark green, with brown gloss and metallic reflection; gaster dark brown. Antenna with scape and pedicel yellowish brown, but brownish in dorsum, other segments of antenna dark brown; mandible yellowish brown and margin of teeth brownish; legs yellowish brown except coxae concolorous with body; fore wing hyaline, wing venation light yellow.

Head in frontal view, 1.27× as wide as high (Fig. 45); frons with very dense reticulation; antennal scrobe with rather large reticulation; lower face flat, with densely transverse striation except lower edge of clypeus smooth; eye height 0.64× head height, eyes separated by 1.23× eye height; scrobe shallow, extending upwards but not reaching anterior ocellus. Antennal insertion slightly above lower ocular line, distance from upper margin of torulus to anterior ocellus 1.58× distance from lower margin of torulus to lower margin of clypeus; clypeal margin protruded, emarginate in middle with two small blunt teeth; oral fossa width 0.59× head width. Head in lateral view (Fig. 44) with malar sulcus inconspicuous, eye height 2.2× malar space. Antennal scape as long as eye height, nearly reaching lower margin of anterior ocellus; length of pedicel and flagellum combined shorter than head width (0.85×); pedicel in lateral view 3× as long as broad; anelli transverse; each funicular segment slightly longer than broad; each funicular segment with one row of longitudinal sensilla; clava slightly clavate, 2.57× as long as broad, micropilosity only limited to apex of third clava segment. Head in dorsal view 2× as wide as long, vertex convex, occipital carina strong; eye length 1.87× temple; POL 1.49× OOL.

Head width 1.31× as broad as thorax. Mesosoma 1.43× as long as broad. Pronotum with coarse reticulation, 0.87× as broad as thorax; pronotum with middle length 0.23× as long as mesoscutum, collar subhorizontal and not margined, posterior margin smooth. Mesoscutum 2× as broad as long, with finely dense reticulation (Fig. 46), posterior reticulation larger than anterior reticulation; notauli incomplete but conspicuous anteriorly. Scutellum flat, as long as broad, frenal line absent; finely reticulate. Propodeum medially 0.43× as long as scutellum, central area flat and with regular reticulation; plicae complete and parallel anteriorly, separated by 1.82× medial length of propodeum; median carina complete; propodeum with short, convex nucha; spiracles elongate, 2× as long as broad, separated by the width of spiracles from hind margin of metanotum; area below spiracles with conspicuous and deep reticulation. Fore wing (Fig. 47) 2.38× as long as broad, without marginal fringe; upper surface densely pubescent; basal vein with sparse setae, basal cell bare, speculum only stretched to the base of marginal vein; upper surface of costal cell bare, lower surface with one complete row of setae and distal 1/3 with two rows of short setae; submarginal vein 2.37× as long as marginal vein; marginal vein 2.64× as long as stigmal vein; postmarginal vein as long as marginal vein; stigmal vein slightly curved.

Petiole invisible dorsally. Gaster long ovate, 2× as long as broad; 0.89× as broad as thorax width, 1.33× as long as length of mesosoma; surface of each tergite coriaceous; Gt_1_ covering 1/3 of gaster, posterior margin straight and with small hollow in middle; posterior margin of other tergites straight; terminal acute.

Male. Body length 1.3-1.9 mm, head and mesosoma black, with yellow-green shine; antennae yellow; legs yellow except coxae concolorous with body. Antennae with long hair, each funicular segment longer than its broad, with long hair on antenna. Gaster ovate, with an oval pale spot between Gt_1_ and Gt_2_.

##### Material examined.

Holotype. China: ♀, Qinghai: Golmud, Guolemude, 2880m, 36.26°N, 94.53°E, 14.IX.2001, leg. Chao-Dong Zhu. Paratype. China: 5♂, 4♀, same data as holotype; 2♀, Qinghai: Delhi, Baingoin, 2900m, 16.IX.2001, leg. Chao-Dong Zhu; 1♀, Qinghai: Qilian, 2790m, 19.IX.2001, leg. Chao-Dong Zhu; 2♀, Qinghai: Dulan, Xiangride, 10.VI.1997, leg. Chao-Dong Zhu; 4♀, Qinghai: Xining, 3-4.VI.1997, leg. Chao-Dong Zhu; 2♀, Qinghai: Tongren, Mailin, 14.VI.1997, leg. Chao-Dong Zhu.

##### Etymology.

The specific name is consist of the spelling of the type locality “qinghai” and the suffix “-*ensis*” represent source.

##### Hosts.

Unknown.

##### Distribution.

China (Qinghai, Yunnan).

## Supplementary Material

XML Treatment for Dibrachys

XML Treatment for Dibrachys (Allodibrachys)

XML Treatment for Dibrachys hians

XML Treatment for Dibrachys kojimae

XML Treatment for Dibrachys koraiensis

XML Treatment for Dibrachys kunmingica

XML Treatment for Dibrachys yunnanensis

XML Treatment for Dibrachys (Dibrachys)

XML Treatment for Dibrachys braconidis

XML Treatment for Dibrachys confusus

XML Treatment for Dibrachys golmudica

XML Treatment for Dibrachys liaoi

XML Treatment for Dibrachys maculipennis

XML Treatment for Dibrachys microgastri

XML Treatment for Dibrachys qinghaiensis
