# Corrigenda: Jiao TY, Yao QY, Xiao H (2017) Review of *Dibrachys* Förster from China (Hymenoptera, Chalcidoidea, Pteromalidae). ZooKeys 656: 123–149.

**DOI:** 10.3897/zookeys.709.20780

**Published:** 2017-10-18

**Authors:** Tian-yang Jiao, Qin-ying Yao, Hui Xiao

**Affiliations:** 1 Key Laboratory of Zoological Systematics and Evolution, Institute of Zoology, Chinese Academy of Sciences, Beijing, 100101, P. R. China; 2 College of Life Sciences, University of Chinese Academy of Sciences, Beijing, 100101, P. R. China; 3 College of Life Science and Technology, Hebei University, Baoding, 071002, P. R. China

In a recent paper we described four new species of *Dibrachys* from China. Unfortunately we failed to specify the depositories of the primary types which render the names unavailable according to Article 16.4.2 of the fourth edition of the International Code of Zoological Nomenclature (ICZN 1999). This short note is to rectify the situation and make the names available. All holotypes and paratypes of the four new species (listed as follows) were deposited in the Zoological Museum, Institute of Zoology, Chinese Academy of Sciences (IZCAS).

***Dibrachys kunmingica* Jiao & Xiao, 2017 (pp 130–132)**

**Holotype.** ♀, China: Yunnan: Kunming, 25.94°N, 102.42°E, IV.1954, leg. Ding-Xi Liao, IOZ(E)1221655. Paratype. 1♀, IOZ(E) 1221656, same data as holotype.

***Dibrachys golmudica* Jiao & Xiao, 2017 (pp 136–137)**

**Holotype.** ♀, China: Qinghai: Golmud, Guolemude, 2880m, 36.26°N, 94.53°E, 14.IX.2001, leg. Chao-Dong Zhu, IOZ(E) 1812507. Paratype. 1♀, IOZ(E) 1812508, same data to holotype; 3♂, 6♀, Inner Mongolia: Ejin B., 11.VI.1981, ex. *Dinorhopala* on *Populus diversifolia*, leg. Hua-Qiang Shao, IOZ(E) 934771-934775, IOZ(E)934777-934779.

***Dibrachys liaoi* Jiao & Xiao, 2017 (pp 137–139)**

**Holotype.** China: ♀, Beijing: Miyun Reservoir, 40.29°N, 116.50°E, 18.VII.1983, ex. pupae of *Dendrolimus tabulaeformis*, leg. Ju-Wen Wu, IOZ(E) 934465. Paratypes: China: 1♂, 2♀, same data as holotype, IOZ(E) 934466-934468; 2♀, Beijing: Miyun Reservoir, 20.VII.1983, ex. pupae of *Dendrolimus tabulaeformis*, leg. Ju-Wen Wu, IOZ(E) 934469-934470; 5♀, Beijing: Huairou, 15.VI.1982, ex. *Illiberis pruni*, leg. Mr. Jin, IOZ(E)934694-934698; 1♀, Beijing: Songshan, 26.VIII.1984, ex. *Illiberis pruni*, IOZ(E)934450; 4♀, Beijing: Changping, 15.VI.1981, ex. *Locastra muscosalis*, leg. Zhen-Hua Liu, IOZ(E) 934471-934474; 1♀, Beijing: Yuanmingyuan Imperial Garden, 18.VII.1984, ex. larvae of *Lymantria dispar*, leg. Mu-Zong Cheng, IOZ(E)934486; 6♀, Beijing: Yuanmingyuan Imperial Garden, 2.VI.1984, ex. larvae of *Lymantria dispar*, leg. Ding-Xi Liao, IOZ(E)934418-934423; 4♂, 5♀, Beijing: Mentougou, late July of 1983, ex. *Prothesia similes xanthocampa*, leg. Sui-Hua Zhao, IOZ(E)934426-934432, IOZ(E)934434-934435; 1♀, Beijing: Qingbaichang, leg. Ding-Xi Liao, IOZ(E)934488.

***Dibrachys qinghaiensis* Jiao & Xiao, 2017 (pp 145–146)**

**Holotype.** China: ♀, Qinghai: Golmud, Guolemude, 2880m, 36.26°N, 94.53°E, 14.IX.2001, leg. Chao-Dong Zhu, IOZ(E) 1812509. Paratype. China: 5♂, 4♀, same data as holotype, IOZ(E) 1812510-1812518; 2♀, Qinghai: Delhi, Baingoin, 2900m, 16.IX.2001, leg. Chao-Dong Zhu, IOZ(E) 1812519-1812520; 1♀, Qinghai: Qilian, 2790m, 19.IX.2001, leg. Chao-Dong Zhu, IOZ(E) 1812521; 2♀, Qinghai: Dulan, Xiangride, 10.VI.1997, leg. Chao-Dong Zhu, IOZ(E) 1812522-1812523; 4♀, Qinghai: Xining, 3-4.VI.1997, leg. Chao-Dong Zhu, IOZ(E) 1812524-1812527; 2♀, Qinghai: Tongren, Mailin, 14.VI.1997, leg. Chao-Dong Zhu, IOZ(E) 1812528-1812529.
